# Carcinoma of the Uterus. The Importance of Early Diagnosis: Symptoms and Treatment

**Published:** 1908-04-04

**Authors:** Frederick Bowreman Jessett

**Affiliations:** Senior Surgeon, Cancer Hospital, Brompton.


### Hospital Clinics.

### CARCINOMA OF THE UTERUS.

### Symptoms and Treatment: the Importance of Early Diagnosis.

By FREDERICK BOWREMAN JESSETT, F.R.C.S., Senior Surgeon, Cancer Hospital, Brompton.

Abstract of a Lecture delivered at the Medical Graduates' College and Polyclinic.

During the twenty-five years I have been surgeon to the Cancer Hospital I have had many hundreds of cases of cancer of the uterus under my observation, and in 90 per cent. the disease was far too advanced when they applied for relief to do anything in the shape of a radical operation. This is a most deplorable condition of things, and one that I think should be remedied, as it constitutes a serious reflection on our profession.

In dealing with diseases of such a terrible nature it is a duty we owe to the public to urge our medical brothers and sisters not to allow any false modesty to interfere between them and their patient, but to insist upon a thorough and proper examination per vaginam of every case wherever the slightest suspicion exists, so that it shall not be laid at the door of the medical profession in the future that through supineness they have allowed their unfortunate patients to drift into that condition where surgical aid is of no avail, and condemned them to that end from which there can be no recall.

For convenience of diagnosis it will be well to divide the uterus into two parts, namely the vaginal portion, including the os and cervix, and the body; of these by far the most commonly affected is the first. When the disease is limited to the os uteri, and it is discovered fairly early, the results of operation, either by vaginal hysterectomy or supravaginal amputation of the cervix, are encouraging.

#### Cancer of the Cervix.

The symptoms of cancer of the vaginal portion and cervical canal are: (1) pain, (2) gastric disturbance. (3) vaginal discharge, (4) hæmorrhage, (5) age, (6) loss of flesh and cachexia.

*Pain.*—The patient often experiences slight pain, not acute, but more what she describes as a "dull wearing" pain, a sense of fullness and weight, accompanied by neuralgic pain after exertion, at the lower part of the abdomen. This is seldom of such a character as to cause the patient to seek advice, as after a night's rest it probably disappears, or is relieved. As the disease progresses pain is intensified, and will be confined to the lower part of the back, but chiefly to the hips. Pain, then, is occasionally an early symptom, and when present should not be treated lightly. In many cases, however, there is a complete absence of pain, or, indeed, of any discomfort.

*Dyspepsia.*—Gastric disturbance is often the earliest symptom, a sense of nausea and perhaps a loss of appetite, accompanied by dyspeptic symptoms, which I have known to have been treated for weeks, and, indeed, months, with little avail, until the patient's attention has been attracted to some vaginal discharge, and on examination malignant disease of the uterus discovered. This symptom is quite in keeping with the gastric disturbance which so frequently is present in pregnancy and in uterine displacement and metritis, and it is important to remember: when a patient consults you suffering from dyspeptic symptoms, it would be always wise to inquire into the regularity or otherwise of the uterine function; very possibly she will then complain of some vaginal discharge, or discomfort at the menstrual period, which would at once attract attention, and lead possibly to a vaginal examination.

*Age* is an important factor, the majority of women who are affected with the diseases under discussion being between the ages of 45 and 55, or just about the menopause, although many cases are met with in younger women. On looking through the number of cases in which I have operated by vaginal and abdominal hysterectomy, or by supra-vaginal amputation, I find 45 per cent. were between the ages of 45 and 55, 34 per cent. between 35 and 45, 13 per cent. were over 55, and 7 per cent. younger than 35 years.

*Discharge.*—Vaginal discharge is a symptom which is invariably present as the disease progresses. Unfortunately women are apt to regard this in a very casual way, whether it be watery or blood stained, and to treat it as an ordinary leucorrhœal discharge of no importance; when present, however, it should be regarded with the greatest suspicion, and after the climacteric it is of the utmost importance, and should never be treated lightly. A careful vaginal examination should be insisted on, not only digitally, but with the speculum, to ascertain whence the discharge comes, from an eroded or ulcerated os, or from the vaginal mucous membrane or the uterine canal. The character of the discharge must also be examined; in the early stages it is slightly purulent or watery, quite different to that found in leucorrhœa; as the disease progresses it becomes more profuse and evil-smelling, and often blood stained. On examination it will be noticed that the diseased surface readily bleeds, or, if limited to the canal, on passing the sound bleeding will follow. Later the discharge increases, becomes more sanious, and smells badly, and is often accompanied by sharp bleeding.

*Hæmorrhage* is a most significant symptom, and is often the earliest indication the patient has that anything is wrong. It frequently is first noticed after coitus, and on examination extensive disease is probably found already to exist. In persons past the climacteric, bleeding must be looked upon with the greatest possible suspicion, and if this is accompanied by discharge and pain I believe it to be absolutely diagnostic of cancer. In younger people there are many causes for menorrhagia or metrorrhagia, but in any cases where bleeding exists a very careful examination must be insisted on. In urging a vaginal examination the importance of it should be pointed out to the patient, as if erosion or ulceration is present it can readily be cured, whereas if left without treatment it is impossible to say what is the real cause of the trouble of which they are complaining; if objection is still taken to the examination the medical attendant would be justified in urging as a reason for doing so that the condition existing, if not attended to, may result in cancer.

### Cancer of the Fundus.

So much, then, for the symptoms of cancer of the os and cervix. When the disease is situated in the fundus, the symptoms are often very obscure. There is ordinarily very little or no pain to indicate mischief, and the discharge in the early stages is not such as to cause alarm; the loss at the menstrual period may be somewhat excessive, but little notice is taken of this. The os and cervix may appear perfectly normal, but on making a vaginal examination the uterus will often be found somewhat enlarged, and the speculum will reveal a slightly purulent discharge exuding from the os, often slightly blood-stained or discoloured. As the disease advances there may be intermittent bleeding between the periods, especially after any excessive sexual excitement, or over-exertion. The os and vaginal portion of the organ appear to be quite healthy; on passing the sound free hæmorrhage sometimes takes place. But it must be recognised that cancer of the fundus or body of the uterus is most difficult to diagnose from symptoms, as many of the same symptoms occur in both acute and chronic endometritis and in tubercular disease.

*Loss of flesh* is a very significant accompaniment, which sooner or later occurs in all cases. As a rule, however, what is usually known as cancerous cachexia does not appear until quite late in the disease, and therefore can scarcely be looked upon as a symptom, but more as a result of the disease.

The diagnosis of cancer of the cervix based only on symptoms may be most confusing, but you will remember that I laid stress upon the points that should arouse your suspicion. But you are not to condemn any patient on symptoms alone; should they be sufficient to cause you to urge a thorough examination in most cases of cancer of the vaginal portion of the uterus or cervix the condition of the parts will suffice to confirm your suspicion. In some cases, however, in the early stages it may not be so easy to differentiate between a cancerous ulcer and ordinary ulceration, or perhaps a syphilitic ulcer; in such cases a small piece must be excised and submitted to examination by an expert pathologist—this can always be done in our day by sending the specimen to one of the pathological research institutions.

When submitting scrapings, etc., for microscopic examination a report of the condition of the patient from whom they have been taken should always be sent. In suspected cases of cancer of the body of the uterus the cervix should be dilated by a laminaria tent, followed by Hegar's dilators, till the finger can be introduced. Soft growth of a whitish colour removed from the body of the uterus is almost always malignant, as is also friable growth in mass and an irregular ulcerated cavity. The curette may be lightly used if but a little growth is found with the finger. The diagnosis in these cases from inflamed hypertrophied endometrium is sometimes difficult unless a portion of the muscular wall be removed with the curette. It is usually a case for a pathological expert, whose opinion, however, should be considered in connection with the clinical features of the case, which in doubtful cases are not less valuable than the microscopic appearances as an aid to diagnosis. These cases of cancer of the body are comparatively rare, and when they exist they may be looked upon as purely local affections, for the glands are not affected until quite late in the disease.

### Treatment.

With respect to treatment there can only be one end to be aimed at, namely, total extirpation of the diseased part. This can be accomplished in very early cases, in which the disease is limited to the vaginal portion of the organ, as has been proved by Sir John Williams and other eminent gynæcologists, and confirmed by my own practice, by high amputation of the cervix. I have, however, discarded this operation for many years in favour of total extirpation of the uterus by the vaginal route, as by the high operation it is impossible to be sure you have got free of the disease. By vaginal hysterectomy I have the satisfaction of knowing that a large number of my patients have been free from any recurrence for a number of years. Undoubtedly in some cases it is wiser to remove the diseased organ by the abdominal route, and it is claimed for this operation that the glands can be removed and any disease which may implicate the ureters be dissected out; however, if the disease has extended so far, recurrence at an early date is certain. The pendulum has in the present age, in my opinion, swung too far over, tending to very extensive operations when lesser ones may answer equally as good a purpose. In comparing the relative merits of the abdominal operations with the vaginal there is still a great discrepancy of opinion, as Besson (*Jour. Sci. Med.*, Lille, June 11-18, 1904) has pointed out; after going very carefully into the question, the mortality at present in cases operated upon is two or three times greater after total abdominal hysterectomy than after vaginal hysterectomy. The proportion of 38 per cent. of survivals in operated cases after two years appears encouraging. The greater proportion of survivals in cases operated on by vaginal hysterectomy indicates the corresponding superiority of this operation in cancers sufficiently localised. It is difficult, then, to lay down any hard and fast rule as to which operation shall be adopted. I think when the fundus or body is attacked it has been pretty well established that no glandular infection takes place unless the disease ulcerates through the wall of the uterus; therefore in such cases vaginal hysterectomy is the better operation, for the reason that shock to the system is not nearly so severe and patients make a much readier convalescence.

Vaginal hysterectomy is also, in my opinion, the better operation for those cases in which the disease is limited to the os and vaginal portion of the uterus. But when the disease has extended up the cervical canal, infiltrating the cervix and possibly infecting the cellular tissue, then the abdominal route, or perhaps the combined abdominal-vaginal operation, would be the wiser, if not the only operation which would hold out any hope of success.

Lastly, those cases in which the uterus is fixed, and the ureters and bladder possibly implicated, I am convinced they are better left rigidly alone, notwithstanding Professor Wertheim's brilliant record.

Within the last few weeks I have seen patients on whom I have operated—some as long ago as 1884, and others at somewhat shorter periods, either by high amputation of the cervix or by vaginal hysterectomy—who are perfectly well, and have never had any recurrence of the disease since the operation. If, then, patients can be freed from their disease by such minor operations as high amputation of the cervix or vaginal hysterectomy when the disease is discovered in its early stage, it will at once be recognised how important it must be to educate women to recognise the significance of symptoms which may point to any abnormal condition connected with the uterine functions. The onus of this must undoubtedly fall to the lot of the medical practitioner, for it is to him that patients of necessity apply in the first instance. I would urge upon you, then, in all cases of abnormal hæmorrhage or discharge to insist upon a vaginal examination—at first digital, afterwards, if necessary, with the speculum. The one great difficulty which has to be contended with is undoubtedly the ignorance of women in understanding the significance of abnormal discharge or pain, and it is a difficult problem to know how to educate them in the matter. I am convinced that many women shirk consulting their medical man from natural but ill-timed modesty, fearing he may wish for an examination; secondly, because they believe the hæmorrhage or discharge they suffer from is due to natural causes or change of life; and lastly, they dread the verdict. What is required is to encourage patients to apply early when suffering from any abnormal symptoms, to impress upon them that in the early stages the disease is curable. This can only be accomplished, in my opinion, by the family medical attendant; it is to him the mother of a family applies in all cases of trouble, and it can be only by the confidence existing between the medical attendant and patient that we can hope to accomplish this very desirable end. Much might be done, no doubt, by the better education of midwives and district nurses, and I was pleased to see at a discussion upon this subject at the annual meeting of the B.M. Association at Exeter that a resolution was passed urging "The Council to appoint a committee to consider the best means of disseminating knowledge of the importance of the early recognition of uterine cancer."

